# CORRELATION BETWEEN TRAUMATIC BRAIN INJURY, OBESITY AND INSULIN-RESISTANCE – A CASE REPORT

**DOI:** 10.2340/jrm-cc.v8.36827

**Published:** 2025-02-03

**Authors:** Laura De WILDE, Charlotte De RUYSSCHER, Kristine OOSTRA

**Affiliations:** 1 Faculty of Medicine, Ghent University, Ghent, Belgium; 2 Department of Physical Medicine and Rehabilitation, University Hospital Brussels, Brussels, Belgium; 3 Department of Physical and Rehabilitation Medicine, Ghent University Hospital, Ghent, Belgium

**Keywords:** brain injuries, traumatic, cognitive dysfunction, diffuse axonal injury, exercise, insulin resistance, metabolic diseases, obesity, rehabilitation

## Abstract

**Introduction:**

Traumatic brain injury is a significant global health concern. It often results from high-velocity accidents and leads to diffuse axonal injury, causing consciousness disorders and potentially permanent cognitive and behavioural changes. Individuals with traumatic brain injury often exhibit weight gain, potentially leading to obesity. This weight increase is influenced by cognitive dysfunction, reduced physical activity and metabolic changes.

**Case report:**

A 23-year-old woman suffered a traumatic brain injury after a traffic accident, with an initial Glasgow Coma Scale score of 5/15. Positive neurological improvement was observed during her stay in the intensive care unit and the neurosurgical department. Subsequently, she was transferred to the rehabilitation department, where she faced significant challenges, including mood fluctuations, cognitive impairments and substantial weight gain. Her body mass index (BMI) increased from 23 kg/m^2^ pre-accident to a maximum of 36 kg/m^2^, despite interventions like medications and lifestyle changes. Blood tests revealed insulin-resistance and metformin initiated a successful weight reduction.

**Conclusion:**

Managing weight gain following traumatic brain injury requires effective interventions that acknowledge its complexity and multifaceted nature, including lifestyle modifications, behavioural therapy and potentially pharmacotherapy or bariatric surgery. Further research is essential to better understand underlying mechanisms and evaluate intervention effectiveness in this specific patient population.

## LAY ABSTRACT

Individuals who had a severe head injury, also known as traumatic brain injury, often experience weight gain during their rehabilitation. In this case, a 23-year-old woman who suffered a traumatic brain injury after a car accident gained substantial weight, starting from a body mass index (BMI) of 23 kg/m^2^ before the accident to a maximum of 36 kg/m^2^ during her rehabilitation. These individuals face challenges in maintaining a healthy diet, managing impulses, engaging in regular exercise and coping with metabolic alterations. It is important for healthcare practitioners to guide and support these patients, taking a comprehensive and tailored approach to address their unique needs.

Traumatic brain injury (TBI) is a significant contributor to global morbidity and mortality, particularly amongst the younger demographic (1, 2). It frequently results from high-velocity vehicle accidents, falls, assaults and sports-related incidents. TBI subjects the brain parenchyma to substantial rotational and translational forces, leading to traumatic axonal damage, commonly referred to as diffuse axonal injury (DAI) (3, 4). Initially, DAI manifests with a broad spectrum of consciousness disorders, frequently causing a reduction in the Glasgow Coma Scale (GCS) score and leading to a comatose state. Over time, DAI can lead to permanent behavioural changes and cognitive impairments (3).

Individuals who experience a TBI often exhibit weight gain, potentially leading to obesity. This weight increase is influenced by multiple factors, including (*i*) cognitive dysfunction and dysexecutive syndrome, (*ii*) diminished physical activity due to the injury and (*iii*) changes in metabolism and endocrine processes (5–10). Obesity poses a global public health concern due to its escalating prevalence, contributing to elevated risks of diverse metabolic, cardiovascular and skeletal co-occurring conditions, as well as cancer. Additionally, it significantly affects psychosocial well-being and is associated with heightened mortality rates (11–20). Consequently, there is a requirement for effective interventions aimed at addressing obesity, taking into account its complexity and multifactorial nature following a TBI. We present a case involving a patient who experienced substantial weight gain following a TBI. This individual underwent a comprehensive evaluation and received diverse treatments as part of their management.

## CASE REPORT

A 23-year-old woman was hospitalized following a traffic accident. Upon arrival at the hospital’s emergency department, her GCS score was 5/15, leading to intubation and sedation. An MRI of the brain was conducted the day after the accident because of delayed awakening, which revealed the presence of DAI. She remained in a coma for a duration of 1 day and was subsequently extubated after 3 days. After spending 1 week in the intensive care unit and 2 weeks in the neurosurgical department, where positive neurological improvement was observed, she was transferred to the rehabilitation clinic. Upon admission in the rehabilitation clinic, she exhibited spontaneous movement in all 4 limbs and could execute motor commands. However, she was apathetic and disoriented, suffered from fatigue disorder, demonstrated apraxia and was severely slowed with hypophonic delayed speech. Furthermore, she sustained several pelvic fractures that were deemed stable, leading to the adoption of a conservative approach. She was prohibited from bearing weight on her feet but was allowed to mobilize within the limits of her pain tolerance.

A follow-up MRI scan, performed approximately 2 months following the accident, showed the reabsorption of subarachnoid blood and the presence of bilateral supra- and infratentorial DAI. Regarding her rehabilitation progress, upon discharge, she was self-sufficient in performing activities of daily living, including self-care and safe transfers and walking. Nonetheless, there were areas of concern related to her overall fitness, strength on the left side, balance and core stability. Furthermore, there had been notable improvements in terms of alertness, reduced distractibility and enhanced memory. However, information processing remained delayed, and her problem-solving abilities were still reduced. On an emotional and behavioural level, her mood fluctuated. She exhibited limited insight into her condition, which occasionally impacted her motivation for therapy. She was discharged from the hospital after a 17-week stay.

After her hospital discharge, she enrolled in an outpatient multidisciplinary rehabilitation program for approximately 8 months, where noticeable improvements were observed in motor skills, flexibility and balance. Regarding executive skills, she could sustain attention when the demands were moderate. However, when tasks became more intricate, she required assistance due to difficulties in maintaining focus and concentration, along with delayed information processing. Emotionally and behaviourally, her mood remained subject to fluctuations, associated with a reduced impulse control.

Throughout the rehabilitation period, a noticeable increase in body mass index (BMI) was evident. Prior to the accident, she maintained an active lifestyle and had a BMI of 23 kg/m^2^. Upon her hospital discharge, her BMI had increased to 25 kg/m^2^. When ending the outpatient multidisciplinary rehabilitation program, her BMI had reached 28 kg/m^2^. During this program, efforts were made to stabilize and reduce this weight gain by testing both topiramate and methylphenidate for approximately 2 months each, but the impact was limited. The hormonal status was investigated, but no post-traumatic pituitary dysfunction was found. The weight gain was attributed to increased impulsivity, reduced physical activity and a lowered basal metabolism.

**Fig. 1:**
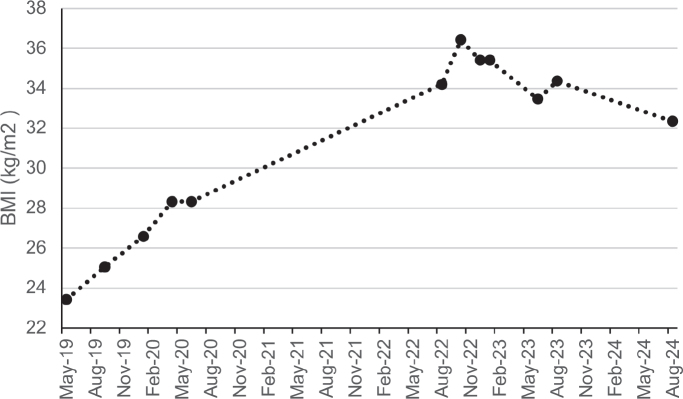
A visual representation of the body mass index fluctuations from the time of the accident to the present.

After completing the outpatient rehabilitation program, the patient was living independently with the necessary support and had secured a part-time job for 16 h per week. However, her BMI continued to increase, finally reaching a maximum of 36 kg/m^2^. Because of this weight gain, she was referred to an endocrinologist for comprehensive assessment and medical guidance. Blood tests revealed insulin-resistance with elevated fasting glucose levels (101 mg/dL) and a normal HbA1c level (5.6%). Consequently, treatment with metformin 850 mg twice a day was initiated. Additionally, there were indications of increased androgens with signs of hirsutism. However, due to her obesity and smoking habits, oral contraception was not prescribed. A sleep study was conducted, revealing mild obstructive sleep apnoea syndrome (OSAS). The patient was advised to resume consultations with a dietitian, and smoking cessation was strongly recommended. A follow-up appointment 6 months later indicated a decrease in BMI to 33 kg/m^2^. Due to abdominal side effects, the metformin dosage was reduced to 425 mg twice a day. The patient is currently under the care of a dietitian and is making efforts to incorporate more physical activity. Unfortunately, her attempts to quit smoking have not been successful. Fig. 1 provides a visual representation of the BMI changes over time.

Finally, the patient recently expressed that she is not doing well. She is experiencing significant physical fatigue, particularly after her work hours. The dysexecutive syndrome is noticeably impacting her daily life, making it challenging for her to tackle both minor and major tasks or seek solutions. To address these issues, additional professional support was recommended to provide administrative and organizational assistance. The patient’s progress is being closely observed, encompassing not only weight management but also her overall well-being.

## REFLECTION

This case illustrates the intricate nature inherent in managing patients with TBI. As mentioned, weight gain in these patients is impacted by various factors, encompassing cognitive impairment, diminished physical activity and metabolic alterations. However, the precise mechanisms behind these phenomena remain incompletely comprehended, as well as their potential interactions.

During the rehabilitation phase, changes in eating habits are expected, leading to potential alterations in weight. These changes can be partly ascribed to the presence of a dysexecutive syndrome. In terms of dietary behaviours, individuals with an executive dysfunction may face challenges related to meal planning and food choices, struggle with emotional dysregulation leading to emotional eating and exhibit difficulties in controlling impulses(5, 21). Hence, a thorough evaluation of each patient’s specific nutritional needs, along with proper support and dietary guidance, should help bypass these issues. Future research should explore how cognitive-behavioural interventions targeting improved planning and impulse control can affect eating habits and weight gain in patients with TBI.

Participating in physical activity not only facilitates weight loss but also positively influences physical, cognitive and psychosocial well-being. However, several factors contribute to why patients with a history of TBI often fail to meet the recommended guidelines, which include 150 min per week of moderate-intensity or 75 min per week of vigorous-intensity aerobic physical activity (22). These barriers can be categorized into 2 groups: (*i*) environmental or facility-related obstacles (e.g. lack of transportation, insufficient accessibility to sporting facilities and cost) and (*ii*) personal barriers (e.g. inherent interest in sports, health concerns, including physical or cognitive disabilities; anxiety; lack of time, motivation, energy or social support; and inadequate know-how). Healthcare practitioners can play a pivotal role in helping patients recognize and overcome these barriers. Additionally, patient education on exactly what physical activity is, and the intensity and amount required for health benefits must be provided. Finally, adapting physical activity programs to align with each patient’s unique needs and limitations is essential (6–9). Future studies should investigate the effectiveness of these personalized physical activity programs.

Metabolic changes after TBI occur in 2 distinct phases: an initial period of pronounced hypermetabolism shortly after the TBI, particularly during the intensive care stay, which poses a severe risk of undernutrition, and subsequently, a recovery phase that may exhibit varying patterns (23, 24). Weight gain during the recovery phase can be attributed in part to the compromised hypothalamus function. The hypothalamus is a critical brain region with a central role in regulating energy balance by modulating appetite, food consumption and managing fat storage and energy expenditure. Hypothalamic injury has been known to be correlated with hyperphagia, which subsequently increases body weight (5, 25, 26). Furthermore, amongst TBI patients, a higher prevalence of insulin resistance was noted compared to the general population (10, 27). One possible explanation is the interference with insulin receptors in the hypothalamus. This central insulin resistance may disrupt glucose metabolism and impair insulin sensitivity in peripheral tissues. Peripheral insulin resistance can consequently hinder the uptake of glucose by cells, causing elevated blood sugar levels and potentially contributing to the onset of type 2 diabetes (27, 28). However, the mechanisms behind this are still the subject of ongoing research. When it comes to addressing insulin-resistant conditions, metformin appears to be the most frequently employed medicine for enhancing insulin sensitivity, reducing hepatic gluconeogenesis and inhibiting glucose absorption in the intestines (26, 29). Whilst pharmacological treatments for obesity are being extensively researched, studies in patients with TBI are lacking (26, 30, 31). One study researched the impact of topiramate, also administered to the patient discussed in this case report, with limited effectiveness. This study included patients with TBI suffering post-traumatic epilepsy and weight gain, including those who were overweight or obese. Topiramate, an antiepileptic drug known to have effects on weight, is sometimes used to treat eating disorders. Notably, positive effects were observed in patients with binge eating disorders, resulting in a significant reduction or complete remission of binge episodes and normalization of body mass index. However, the findings in patients without binge eating disorders were less conclusive (32). Another study on methylphenidate administration in paediatric TBI patients with persistent attention problems demonstrated significant weight loss compared to the placebo, likely due to the known appetite-related side effects associated with the medication (33). Finally, though bariatric surgery has proven effective for obesity, positively impacting factors such as neurological functioning, its application to TBI patients remains unexplored (16, 18–20). Additional research exploring the metabolic changes post-TBI could guide tailored dietary interventions. Studies focusing on metabolic assessments and nutritional modifications, such as meal timing and composition, could provide valuable insights.

In conclusion, addressing weight gain and obesity in patients following a TBI necessitates effective interventions that acknowledge its complexity and multifaceted nature. Recognizing that each contributing factor – executive dysfunction, limited physical activity and metabolic alterations – necessitates specific interventions, it is essential to integrate these components into a standardized treatment program tailored to the individual patient’s needs. This should be approached in a multidisciplinary manner, including lifestyle modifications, behavioural therapy, pharmacotherapy and/or bariatric surgery. Future research should focus on studies that integrate cognitive, physical and metabolic assessments to provide a comprehensive understanding of the interplay between these factors. Additionally, studies examining the combined effects of cognitive-behavioural therapy, personalized physical activity programs and nutritional modifications on mitigating weight gain post-TBI would be valuable. Such research could inform individualized treatment plans and contribute to the development of standardized evidence-based guidelines for managing weight gain in TBI patients.
